# Electrospun patches to deliver combination drug therapy for fungal infections

**DOI:** 10.3389/fddev.2024.1458009

**Published:** 2024-09-16

**Authors:** Karolina Dziemidowicz, Mark Meszarik, Jacopo Piovesan, Mazna A. Almatroudi, Gareth R. Williams, Sudaxshina Murdan

**Affiliations:** Department of Pharmaceutics, UCL School of Pharmacy, University College London, London, United Kingdom

**Keywords:** electrospinning, patch, drug combination, fungus, infection

## Abstract

Fungal infections, though affecting healthcare globally, receive insufficient attention in clinical and academic settings. Invasive fungal infections, particularly caused by combat wounds, have been identified as a critical threat by the US Department of Defense. Monotherapy with traditional antifungals is often insufficient, and so combination therapies are explored to enhance treatment efficacy. However, systemic combination treatments can result in severe adverse effects, suggesting the need for localised delivery systems, such as drug-loaded electrospun patches, to administer antifungals directly to the infection site. This proof-of-concept study hypothesised that dual amorolfine and terbinafine therapy slowly releasing from electrospun patches would be an effective way of eradicating *Candida albicans* when the patch was applied directly to the fungal colony. The feasibility of creating electrospun materials loaded with amorolfine and terbinafine for combination antifungal therapy was investigated. Electrospinning was used to fabricate polycaprolactone (PCL) patches with varying drug loadings (2.5%, 5%, and 10% w/w) of amorolfine and terbinafine either individually or in combination. The incorporation of both drugs in the fibres was confirmed, with the drugs predominantly in an amorphous state. Results showed that combination therapy patches had a significantly greater and prolonged antifungal effect compared to monotherapy patches, with larger zones of inhibition and sustained efficacy over at least 7 days. This study therefore demonstrates that PCL-based electrospun patches containing amorolfine and terbinafine provide superior antifungal activity against *C. albicans* compared to monotherapy patches. This approach could lower required drug doses, reducing adverse effects, and enhance patient compliance due to prolonged drug release, leading to more effective antifungal therapy.

## Introduction

Fungal infections are generally neglected both in the clinic and academic research, despite their high global impact (https://gaffi.org/). The US Department of Defence identifies combat wound infections due to invasive fungi as a high priority emerging threat (Turnbull et al., 2021). *Candida* species are the most prevalent in the wound mycobiome while species belonging to the *Curvularia*, *Malessezia*, *Aureobasidium*, *Cladosporium*, *Ulocladium*, *Engodontium* and *Trichtophyton* genera can also be found (Dowd et al., 2011). Monotherapy with traditional antifungal agents is often inefficient (Baran et al., 2007), and combination therapies are therefore being explored to provide additive or synergistic antifungal effects (Zhu et al., 2023). However, effective, long-term systemic combination therapy with antifungals can lead to adverse effects. There is therefore a strong need for localised delivery systems administering antifungal therapy directly to the site of action, for example, through a drug-loaded wound dressing. Such dressings can be prepared using electrospinning, a fabrication method which allows for the formation of drug-loaded fibrous materials with appropriate handling properties for implants and wound dressings. Electrospinning of drug-loaded patches has been extensively described for a variety of healthcare applications (Dziemidowicz et al., 2021a; Kellaway et al., 2024), with recent focus on antimicrobial treatments (Akombaetwa et al., 2022; Zhang et al., 2024). While there are published examples of electrospun materials with antifungal properties, such as fluconazole-loaded mucoadhesive patches for vaginal candidiasis (Sharma et al., 2016) or poly (lactic acid)-based carvacrol-releasing membranes (Scaffaro et al., 2018), the field remains largely unexplored.

This study therefore explores the feasibility of fabricating electrospun materials loaded with two therapeutic agents (amorolfine and terbinafine) for antifungal combination therapy. These candidates were incorporated into poly (ε-caprolactone) (PCL) electrospun fibres, either alone or in combination, and their antifungal activity assessed in an extended disk diffusion assay against the pathogen most commonly found in fungal wound infections, *Candida albicans.* To the best of our knowledge, this is the first report of the development of electrospun fibres as carriers for antifungal drug combinations.

## Experimental

### Materials and methods

Preparation of electrospun patches. PCL fibres containing no drug (Blank) were prepared by electrospinning a solution of 12% w/v PCL (Mw ∼80 kDa; Sigma-Aldrich) in 2,2,2-trifluoroethanol (TFE; Sigma-Aldrich). This concentration was selected based on the literature and our previous experience of working with PCL (Dziemidowicz et al., 2021b; Dziemidowicz et al., 2023). Solutions were carefully loaded into 5 mL disposable syringes and dispensed through a 21G stainless steel needle connected to a high-voltage direct-current power supply (HCP 14-20000, FuG Elektronik GmbH). Drug-loaded electrospinning solutions were prepared by adding antifungals (amorolfine, terbinafine or a 1:1 w/w mixture of both) directly to the polymer solution and stirring for another 60 min prior to electrospinning. The electrospinning solutions were dispensed at a flow rate of 1.8 and 1.3 mL/h for blank and drug-loaded formulations, respectively. The fibres were collected on baking paper placed on top of a 14.7 cm × 20 cm metal plate collector connected to the grounded electrode. The distance from the needle to the collector was 14 cm. The experiments were conducted at 25°C ± 2°C and relative humidity 35% ± 10%. Full details of the processing parameters are given in Table 1.

**TABLE 1:** Summary of formulation composition and electrospinning parameters used to fabricate antifungal patches.

Formulation name	Drug content	Drug loading (%w/w)	Voltage range (kV)
Blank	No drug	N/A	8–10
Amorolfine 2.5%	Amorolfine	2.5	13–15
Amorolfine 5%	Amorolfine	5	13–16
Amorolfine 10%	Amorolfine	10	16–18
Terbinafine 2.5%	Terbinafine	2.5	16–19
Terbinafine 5%	Terbinafine	5	15–16
Terbinafine 10%	Terbinafine	10	14–17
Mix 2.5%	Amorolfine and terbinafine (1:1)	2.5	15–17
Mix 5%	Amorolfine and terbinafine (1:1)	5	16–18
Mix 10%	Amorolfine and terbinafine (1:1)	10	17–19

Note: Drug loading (%w/w) for the ‘Mix’ formulations refers to the total drug load i.e., of the two drugs together.

The fibres were then analysed by scanning electron microscopy (SEM), Fourier-transform infrared (FTIR), X-ray diffraction (XRD), and differential scanning calorimetry (DSC) and thermogravimetric analysis (TGA). Full experimental details as well as characterisation data for monotherapy fibres are provided in the Supplementary Material.

### Antifungal testing

*Candida* *albicans* (*C. albicans*) cultured on Sabouraud Dextrose Agar (SDA) plates was used to prepare the fungal inoculum. The colonies were swabbed using a sterile inoculating loop and suspended into 5 mL of 0.85% w/v saline in a sterile vial. The absorbance of the suspension was measured using a UV-visible spectrometer (Biochrom Libra S12) at a wavelength of 530 nm and adjusted by either adding additional saline or fungal colonies until the suspension turbidity was in compliance with a 0.5 McFarland standard measured at the same wavelength.

The duration and potency of the antifungal effect of the electrospun patches was established using the disc diffusion method. The electrospun patches were cut into discs (6 mm in diameter) using a metal hole punch and sterilised using UV irradiation within a laminar flow hood for 20 min on both sides. Once the fungal inoculum was spread on the SDA plate, electrospun patch discs were transferred onto the dish using sterile tweezers and gently pressed down. For each plate used, a blank formulation (no drug loaded) was used as a negative control. The plates were then incubated at 32°C for 24 h, after which a photograph of each plate was taken. The electrospun discs were then moved onto a freshly inoculated plate each day to test the duration of drug release and the antifungal effect of the formulations. Using a ruler the zone of inhibition (ZOI) was measured by placing the end of the ruler at the centre of the drug-loaded disc and reading the distance at which the first fungal colonies on the plate were observed. Each formulation was tested in triplicate, and the results are presented as mean ± SD.

Data were statistically analysed using two-way analysis of variance (ANOVA) followed by Tukey’s multiple comparisons test and t-tests. Differences were considered significant when *p < 0.05*. GraphPad Prism version 9.5.00 (GraphPad Software, United States) was used for statistical analysis.

## Results and discussion

**FIGURE 1:**
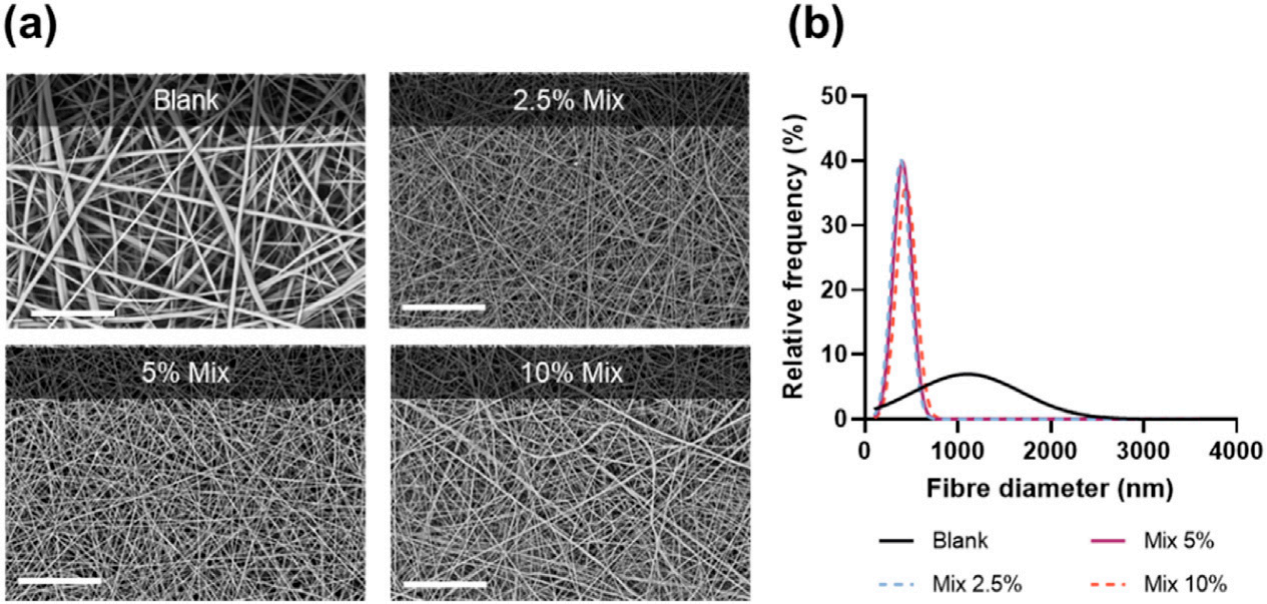
Scanning electron micrographs **(A)**; and fibre diameter size distribution curves **(B)** for unloaded PCL fibres (blank) and combination therapy patches at different drug loadings (Mix 2.5%, 5%, and 10%). Scale bar = 80 µm.

A simple method of blend electrospinning was selected to facilitate future scaled-up manufacture. In this approach, each drug individually or a 1:1 drug mixture was directly added into a polymer solution containing organic solvent. Owing to its well-described biodegradability, biocompatibility and favourable mechanical properties, PCL was chosen as the polymer carrier. As a hydrophobic polymer with slow degradation rate, PCL would be a suitable choice for an extended-release delivery system of poorly soluble drugs such as amorolfine and terbinafine. Moreover, both drugs and the polymer dissolve readily in the electrospinning solvent (TFE), ensuring appropriate physicochemical stability of the electrospinning solution throughout the fabrication process. The electrospinning of the polymer-drug solutions was stable, required minimal optimisation and resulted in the formation of uniform, defect-free, cylindrical fibres (Figure 1A; Supplementary Figure S1.

Fibre diameter measurements (Figure 1B) showed a drastic decrease in fibre diameter upon addition of the drugs, from 1235 ± 639 nm (Blank) to 395 ± 92 nm (Mix 2.5%), 423 ± 128 nm (Mix 5%) and 462 ± 140 nm (Mix 10%). A lower diameter for drug-loaded electrospun fibres is a common phenomenon often attributed to increased conductivity of drug-polymer solutions (Zeng et al., 2003; Zamani et al., 2010; Krogstad and Woodrow, 2014).

**FIGURE 2:**
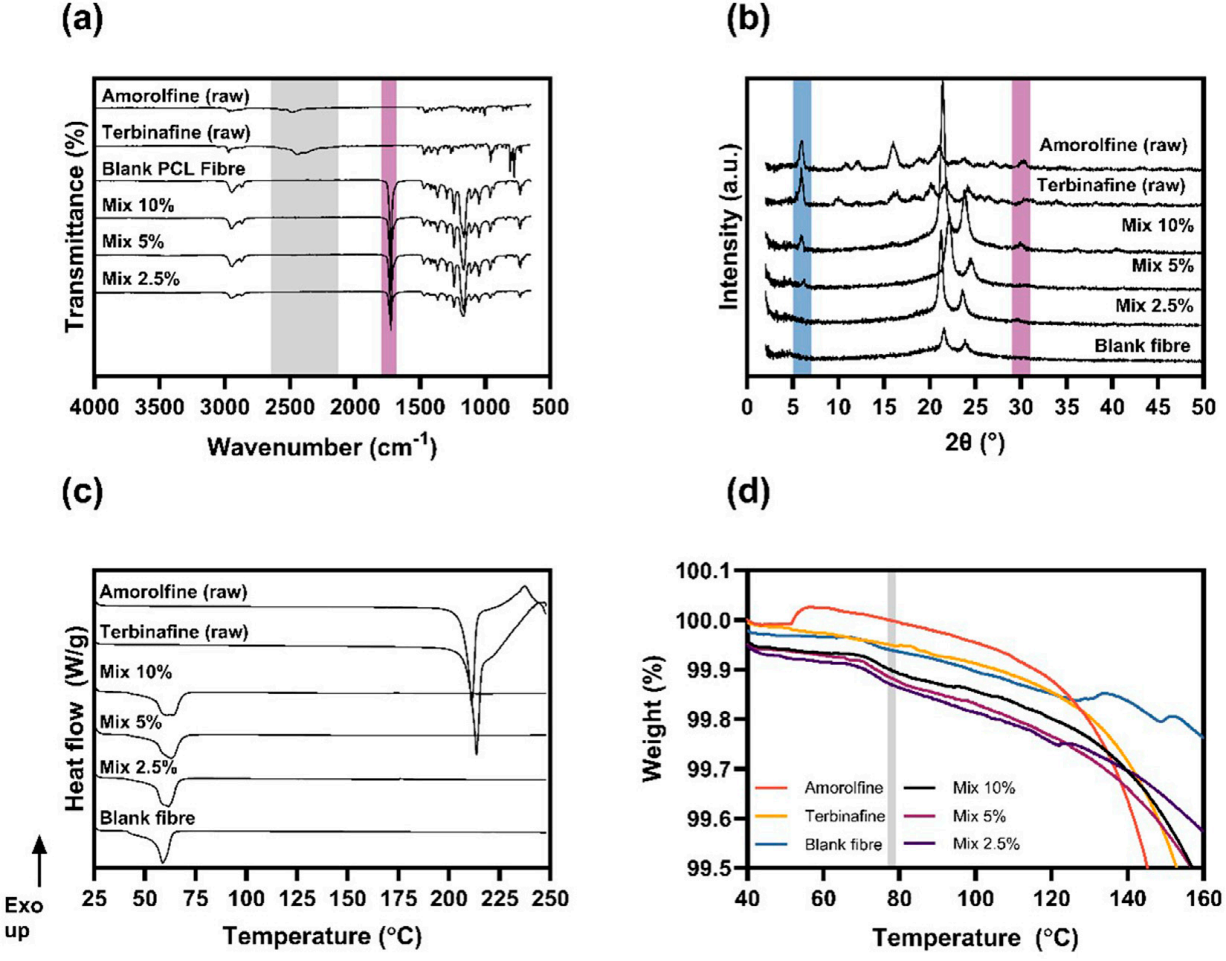
Physicochemical characterisation. FTIR **(A)**, XRD **(B)** and DSC **(C)** data confirm the mostly amorphous nature of amorolfine and terbinafine within the electrospun patches. The TGA analysis **(D)** around the boiling point of TFE (shaded in grey) showed no residual solvent present in electrospun patches.

Monoaxial electrospinning of drug-polymer blends is particularly attractive for the processing of poorly soluble crystalline materials as it typically leads to the creation of amorphous solid dispersions (ASD), therefore enhancing bioavailability. FTIR spectra (Figure 2A; Supplementary Figure S2) were acquired to investigate the chemical composition of the fabricated formulations.

In amorolfine raw material, the characteristic NH^+^ stretching vibration of the nitrogen within the morpholine ring is present at around 2480 and 2560 cm^−1^. Similarly, terbinafine raw material exhibits an amine peak at 2448 cm^−1^ (Kuminek et al., 2013). The FTIR spectrum of the blank fibres is identical to that of previously reported raw PCL (Chiu et al., 2020). In the drug-loaded formulations, neither amorolfine nor terbinafine peaks were observed. In XRD (Figures 2B, Supplementary Figure S3, S4), sharp Bragg reflections observed for the amorolfine and terbinafine raw materials suggest a crystalline structure of both active ingredients (Thatai and Sapra, 2018). Blank fibres containing only PCL have two broad Bragg reflections, consistent with its nature as a semi-crystalline polymer (Chiu et al., 2020). While the XRD patterns of formulations with the lowest drug loading (Mix 2.5%) resembled that of the blank formulation, a small reflection at 5.9° and a broad peak at 29.8° were detected with Mix 5% and Mix 10%, indicating a small amount of crystalline drug in the fibres. A similar observation was made for terbinafine monotherapy patches (Supplementary Figure S3). DSC analysis (Figure 2C; Supplementary Figure S5) shows sharp melting endotherms for the raw drugs (at 210°C for amorolfine and 213°C for terbinafine), consistent with the literature (Thatai and Sapra, 2018; Kerai et al., 2015; Abd-Elmonem et al., 2023) and confirming a crystalline structure. In both cases, this is followed by an exotherm likely arising from drug degradation. Blank PCL fibres also show a melt, at ca. 60°C. The PCL melt is clearly visible in the drug-loaded fibres (both mono- and mixed), but the distinct drug melting endotherms were not present in any of the fibre formulations, confirming the mostly amorphous nature of the drug-loaded electrospun patches. Additionally, TGA analysis (Figure 2D) was performed to investigate whether residual solvent was present in the materials post-fabrication. The unchanged mass in the TGA curve at 78°C (boiling point of TFE) suggests that the solvent had completely evaporated during the electrospinning process.

**FIGURE 3:**
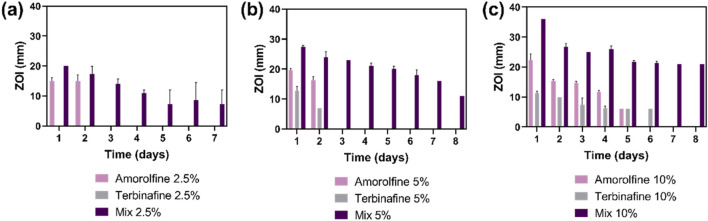
Antifungal effect of electrospun patches presented as zone of inhibition (ZOI) of *C. albicans* growth in a disc diffusion test. The drug combination formulations gave a larger ZOI occurring over a longer period of time following transfer of patches to freshly fungus-inoculated agar plates, showing a more potent antifungal effect than single-drug formulations (amorolfine and terbinafine) at all tested concentrations (2.5% **(A)**, 5% **(B)**, 10% **(C)** w/w drug: polymer). Sustained drug release is evidenced by prolonged antifungal effects visible over at least 8 days. Two-way ANOVA showed a significant difference between monotherapy (either amorolfine or terbinafine) and combination therapy (Mix) for 5% and 10% drug loadings at all tested timepoints (*p < 0.05).* Data are presented as mean ± SD, n = 3.

Antifungal performance was assessed against *C. albicans*, one of the most prevalent fungi in chronic wounds (Dowd et al., 2011; Chellan et al., 2010). Monotherapy patches containing amorolfine or terbinafine only were used as controls. While amorolfine-only patches showed some antifungal activity, terbinafine-only patches were generally ineffective against *C. albicans*, reflecting the known lower activity of terbinafine and previous literature reporting no growth inhibition in *C. albicans* upon treatment with terbinafine-loaded PCL/gelatin fibres (Paskiabi et al., 2017). In contrast, the Mix patches were very effective and clear ZOIs were seen for the Mix formulations at all loadings (2.5%, 5%, 10%) (Figure 3; Supplementary Tables S1-S3), suggesting a potential additive or synergistic effect of amorolfine and terbinafine. This agrees with a recent study by Schaller et al., who explored these two agents in combination against pathogenic fungi in onychomycosis and confirmed their additive effect against *T. rubrum* as well as *T. interdigitale*, encouraging further exploration of electrospun antifungal combination patches for applications in fungal nail infections.

The ZOI could be correlated to the concentration of terbinafine/amorolfine, where Mix 10% achieved the highest ZOI, followed by Mix 5% and Mix 2.5%. For higher drug concentrations (5% and 10%), the combination patches showed significantly (*p* < 0.05) higher ZOIs than for monotherapy at all tested timepoints. Combination patches also showed prolonged antifungal effect over at least 7 days.

## Conclusion

Combination antifungal PCL-based patches containing both amorolfine and terbinafine were fabricated using blend electrospinning. Morphological and physicochemical characterisation confirmed successful incorporation of both drugs within the fibre meshes, and their predominantly amorphous state in the fibres. The effect of combination therapy with amorolfine and terbinafine was demonstrated against *C. albicans*, where combination formulations achieved greater and prolonged inhibition of fungal growth over at least 7 days compared to single drug-loaded patches containing the same total drug concentration. In practice, enhanced antifungal activity could enable the use of lower drug doses, reducing/eliminating adverse effects, while prolonged antifungal activity would reduce the frequency of medicine administration, which could lead to improved patient compliance and thereby to more successful therapy.

## Data Availability

The raw data supporting the conclusions of this article will be made available by the authors, without undue reservation.
