# Supplementary material for: Electrospun patches to deliver combination drug therapy for fungal infections
Source: Front Drug Deliv. 2024 Sep 16;4:1458009. doi: 10.3389/fddev.2024.1458009 (PMC12363251; doi:10.3389/fddev.2024.1458009)
Supplement: Supplementary file 1 [file DataSheet1.docx]

**Supplementary information for**

**Electrospun patches to deliver combination drug therapy for fungal infections.**

Karolina Dziemidowicz, Mark Meszarik, Jacopo Piovesan, Mazna Almatroudi, Gareth R. Williams, Sudaxshina Murdan
Department of Pharmaceutics, UCL School of Pharmacy, 29-39 Brunswick Square, London, WC1N 1AX

1. **Experimental methods**

**Scanning electron microscopy (SEM)**

A sample of approximately 0.5 cm × 0.5 cm was cut from each fibre formulation. The samples were mounted onto aluminium stubs (TAAB Laboratories) with carbon-coated adhesive tabs and sputter-coated with 20 nm gold for 5 minutes (Q150R coater, Quorum) in an argon atmosphere, and analysed with a cerium hexaboride thermionic filament scanning electron microscope (Phenom Pro, Thermo) connected to a secondary electron detector. The diameter of the fibres was calculated using ImageJ software version 1.52a (National Institutes of Health) with a minimum sample size of 100, from three SEM images. The size distribution curves were prepared using Prism software version 8.4.2 (GraphPad).

**Fourier-Transform Infrared (FTIR) spectroscopy**

FTIR spectra were obtained using a Spectrum 100 spectrometer (Perkin Elmer). The spectral data were analysed with the Prism software version 8.4.2 (GraphPad). Data were collected over the wavenumber range from 650-4000 cm^-1^, with resolution 1 cm^-1^ and 4 scans obtained per sample.

**X-ray diffraction (XRD)**

XRD patterns of the samples and reference materials were obtained using a Miniflex 600 (Rigaku) diffractometer supplied with Cu-Kα radiation (λ= 1.5418 Å). A glass sample holder was used. The patterns were recorded in the 2Ө range of 3 - 50º at a speed of 0.5º min^-1^. The generator voltage was set at 40 kV and the current at 15 mA. Data were visualised using Prism software version 8.4.2 (GraphPad).

**Differential scanning calorimetry (DSC)**

Analysis was conducted using a Q2000 DSC (TA Instruments). A small amount of sample was placed inside a non-hermetically sealed aluminium pan (T130425, TA Instruments). DSC analysis was carried out from 0 - 250 ºC at a temperature ramp of 10 ºC/min. Oxygen-free nitrogen gas at a purge rate of 50 mL/min was supplied to the furnace throughout the experiment. Data analysis was carried out using the TA Universal Analysis software version 4.5 (TA Instruments). Prism software version 8.4.2 (GraphPad, USA) was used to plot thermograms.

**Thermogravimetric analysis (TGA)**

Thermogravimetric analysis (TGA) was performed on a Discovery instrument (TA Instruments, Waters LLC). Roughly 3 mg of fibre sample was loaded into an aluminium pan and heated from 40 to 300 °C at 10 °C/min under a nitrogen flow of 25 mL/min. Data were recorded using the Trios software and analysed with TA Universal Analysis.

1. **Results**


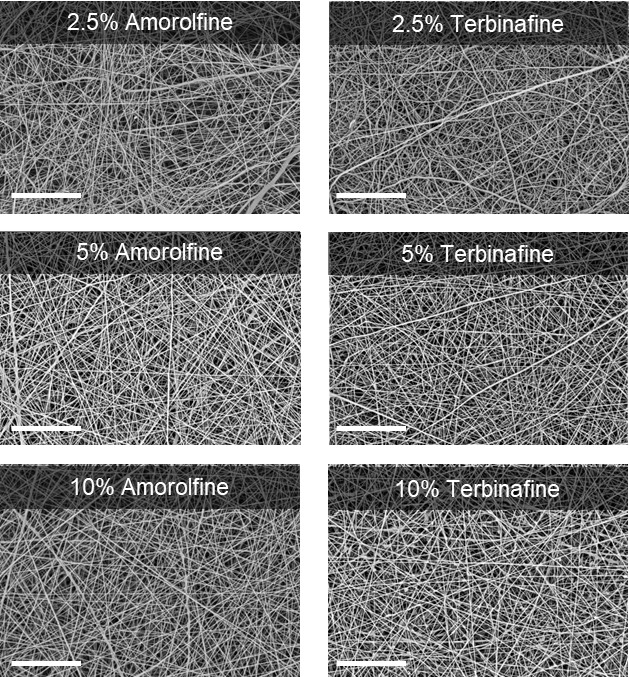


Figure S1: Scanning electron micrographs of electrospun fibres of control single-drug patches containing either amorolfine or terbinafine. Scale bar = 80 µm.


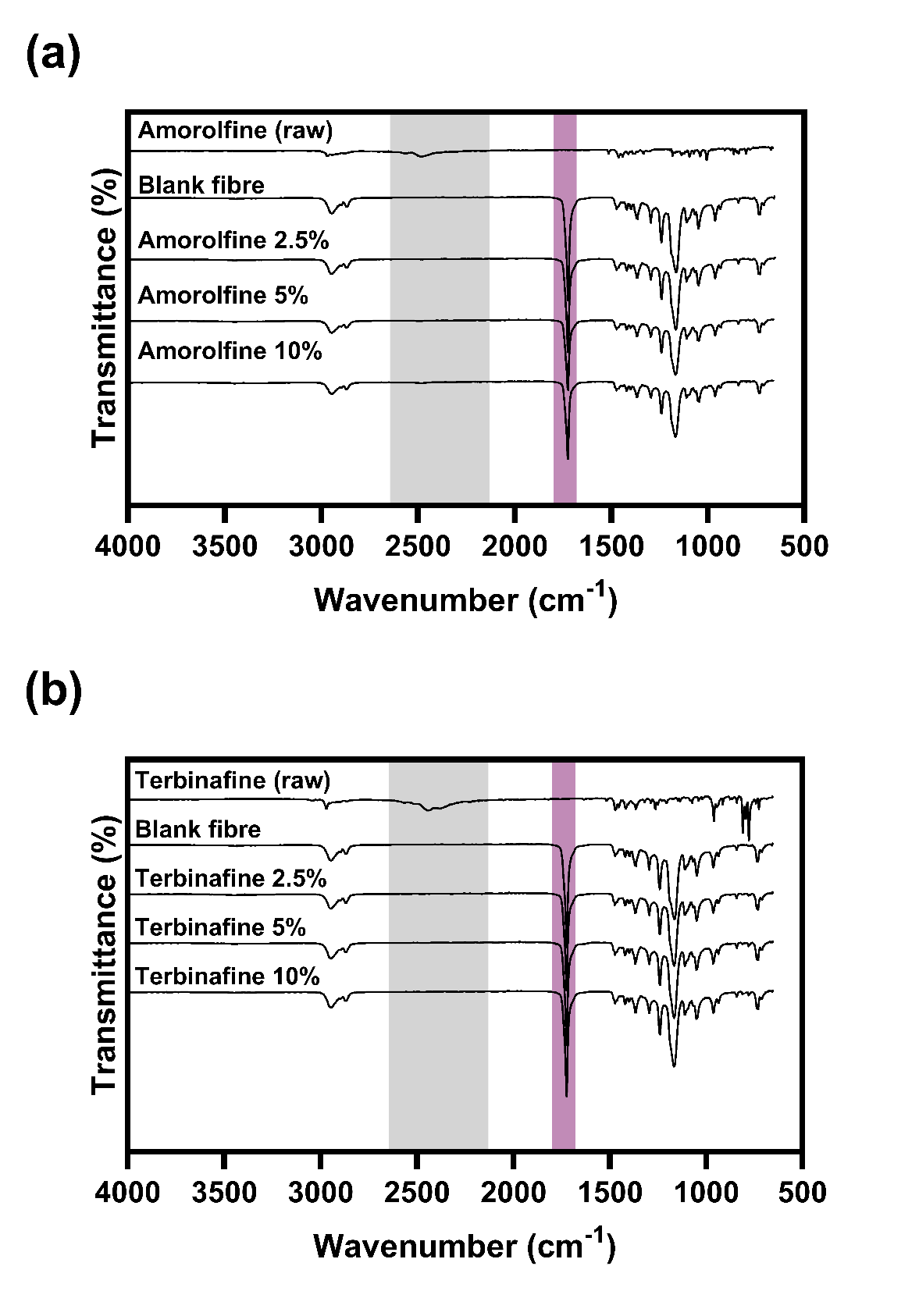


Figure S2: FTIR spectra for monotherapy patches containing either amorolfine (a) or terbinafine (b).


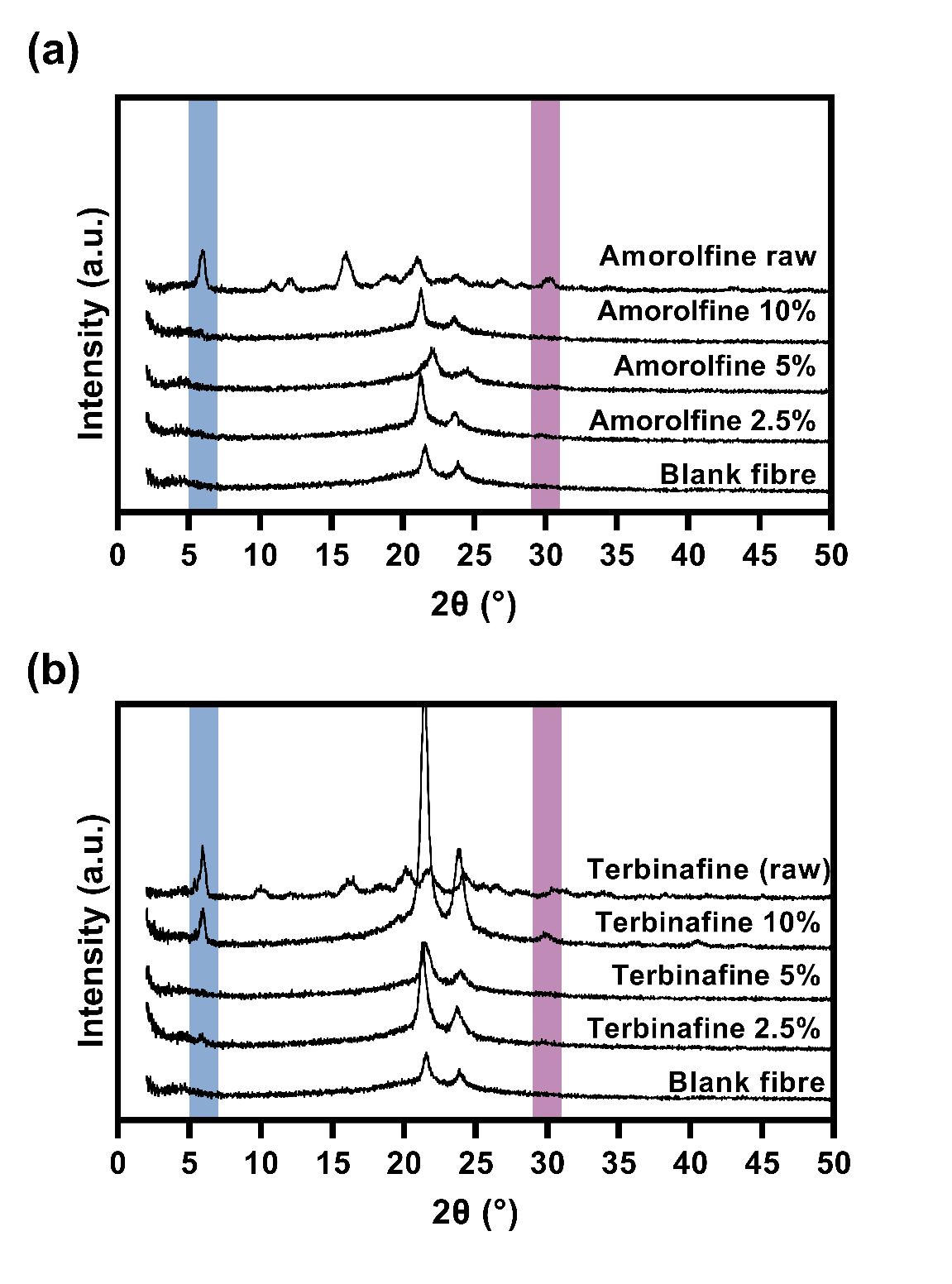


Figure S3: XRD data for monotherapy patches containing either amorolfine (a) or terbinafine (b).

Figure S4: Indexed XRD data for terbinafine raw material (a) and PCL blank fibre (b). Amorolfine raw material XRD data (c) could not be indexed due to the lack of published values.


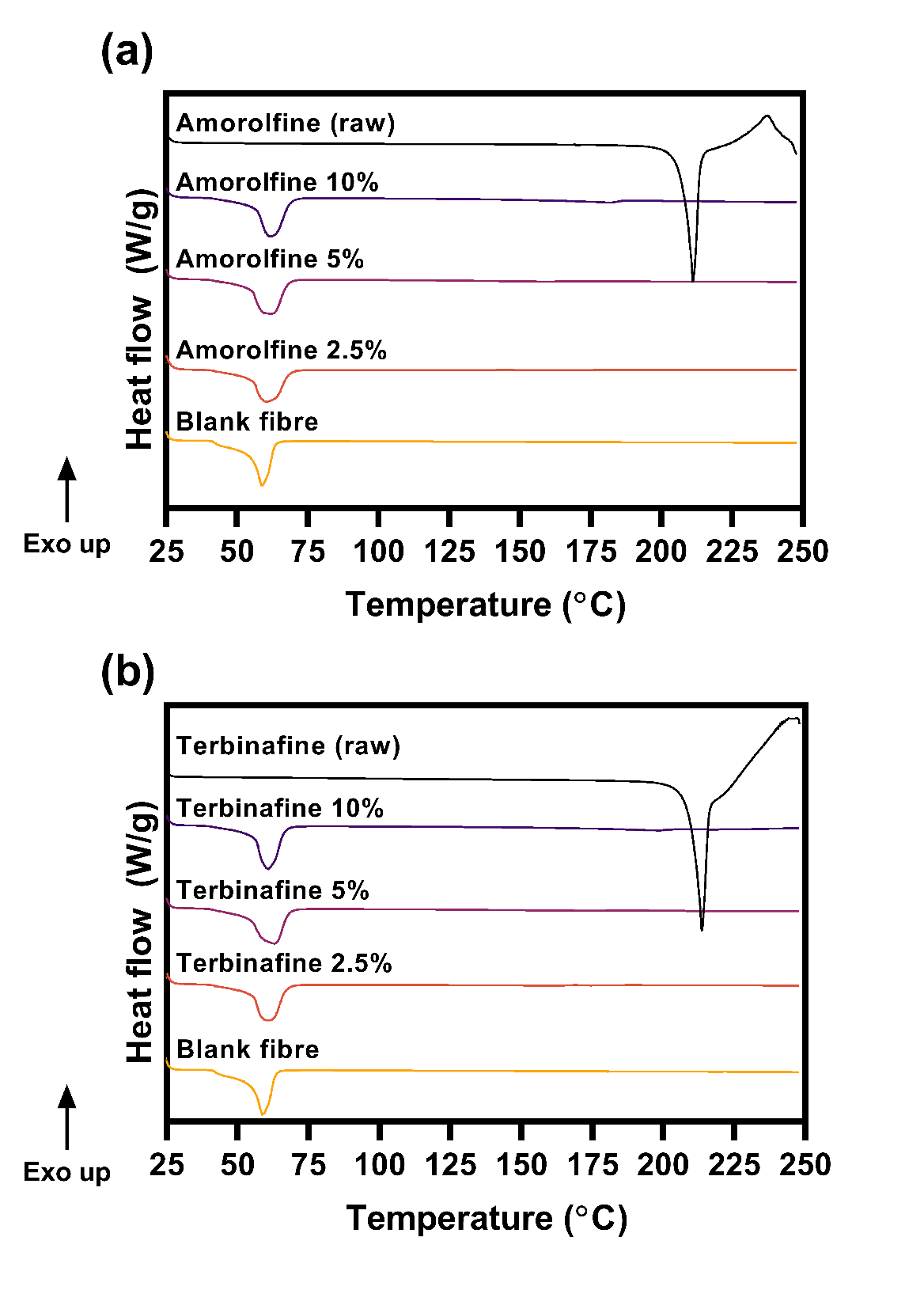


Figure S5: DSC data for monotherapy patches containing either amorolfine (a) or terbinafine (b).

Table S1: Photographs of disc diffusion test in C. albicans for samples at 2.5% w/w drug loading.

|  | Day 1 | Day 2 | Day 3 | Day 4 | Day 5 | Day 6 | Day 7 |
| --- | --- | --- | --- | --- | --- | --- | --- |
| Amorolfine 2.5% | 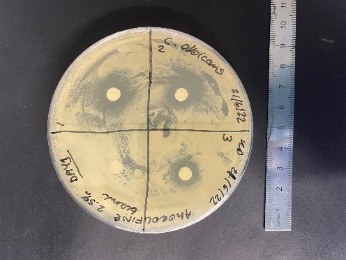 | 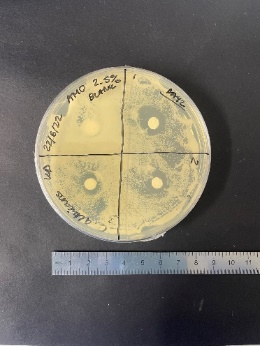 | 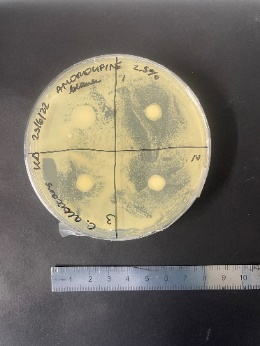 | 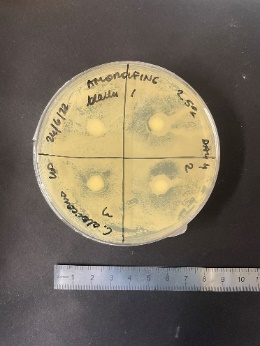 | 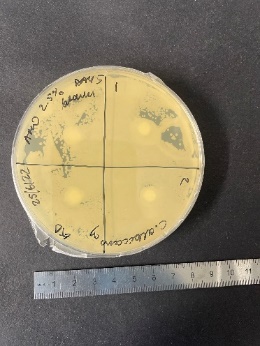 | 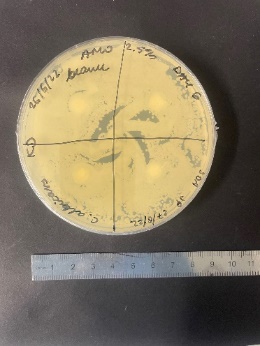 | Terminated |
| Terbinafine 2.5% | 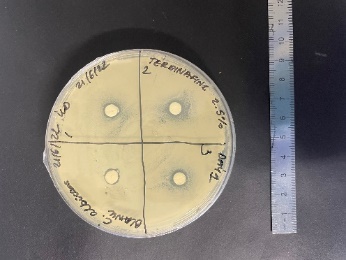 | 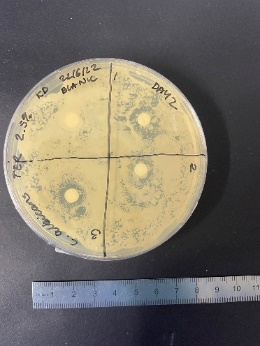 | 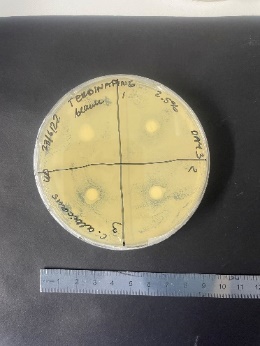 | 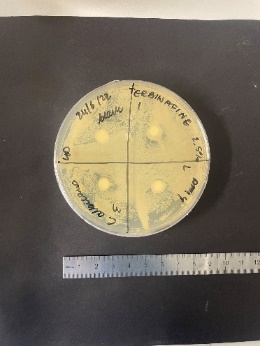 | 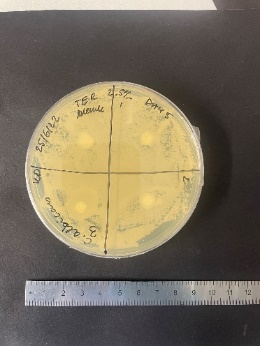 | 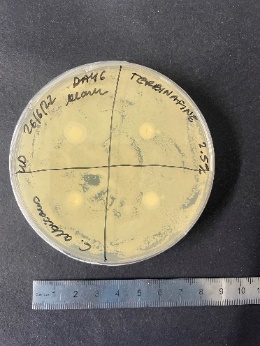 | Terminated |
| Mix 2.5% | 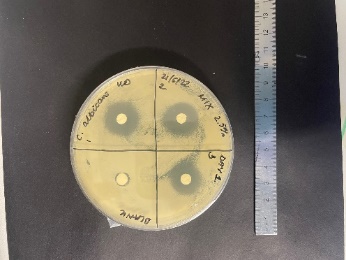 | 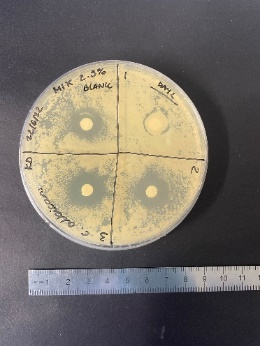 | 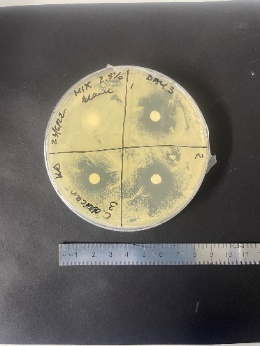 | 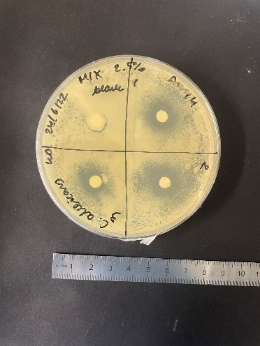 | 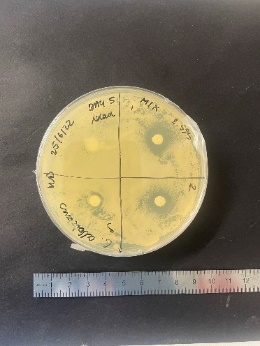 | 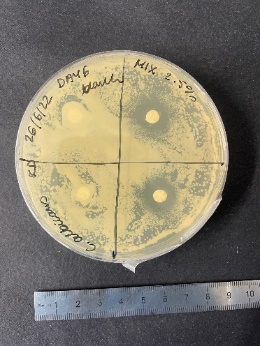 | 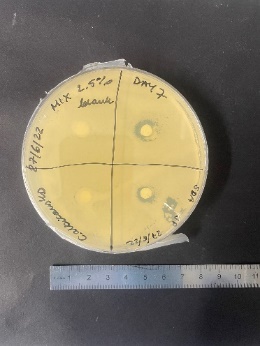 |

*Table S2: Photographs of disc diffusion test in* C. albicans *for samples at 5% w/w drug loading.*

|  | Day 1 | Day 2 | Day 3 | Day 4 | Day 5 | Day 6 | Day 7 |
| --- | --- | --- | --- | --- | --- | --- | --- |
| Amorolfine 5% | 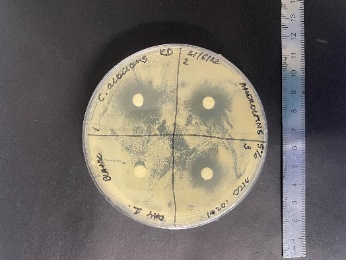 | 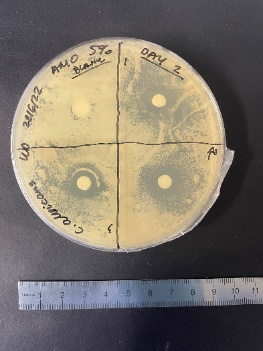 | 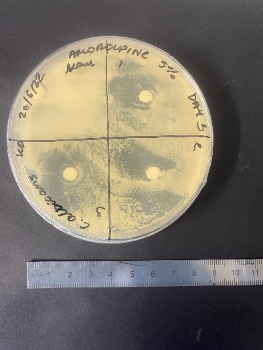 | 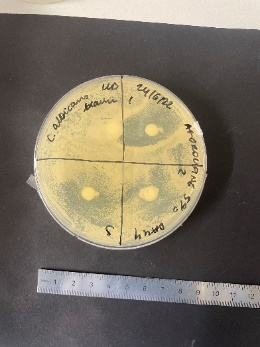 | 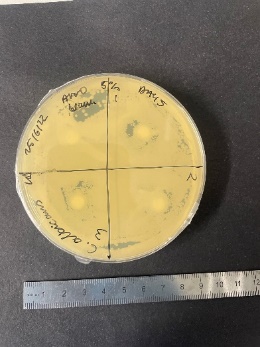 | 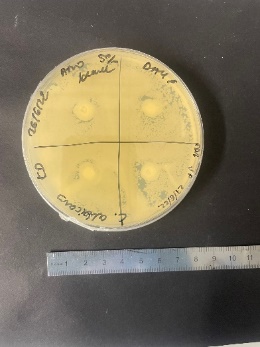 | Terminated |
| Terbinafine 5% | 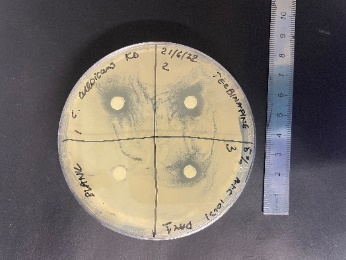 | 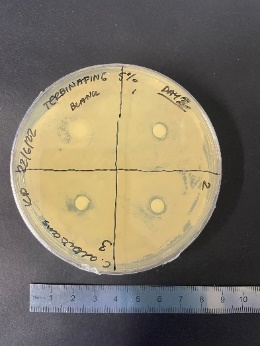 | 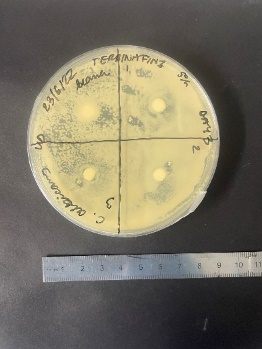 | 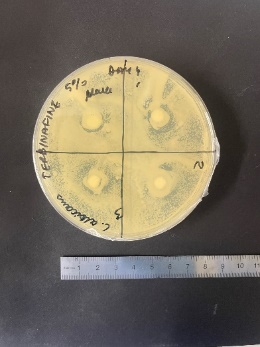 | 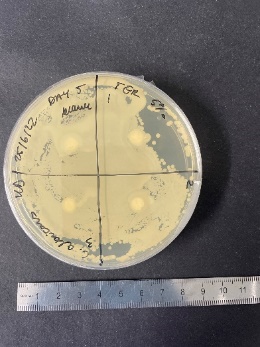 | 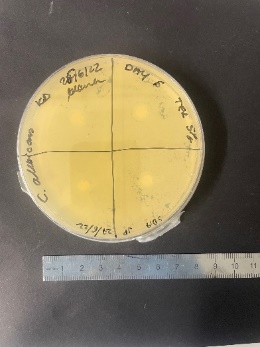 | Terminated |
| Mix 5% | 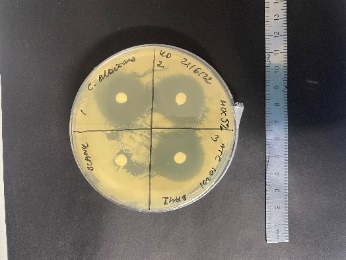 | 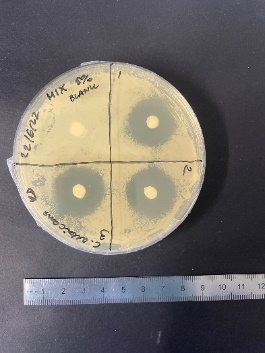 | 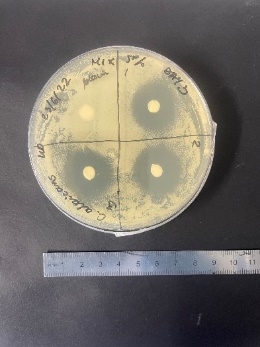 | 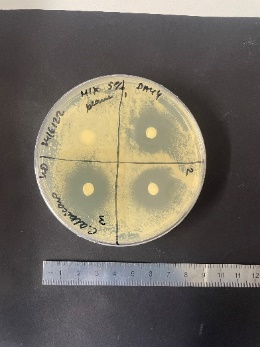 | 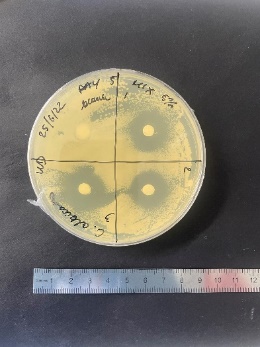 | 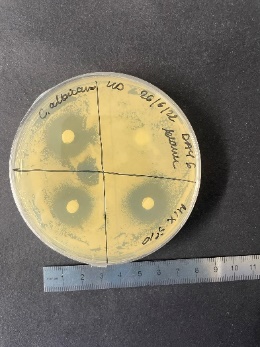 | 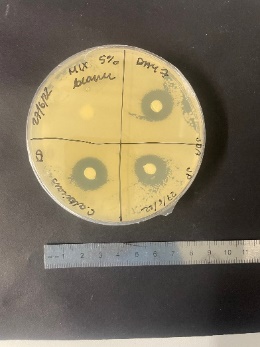 |

*Table S3: Photographs of disc diffusion test in* C. albicans *for samples at 10% w/w drug loading.*

|  | Day 1 | Day 2 | Day 3 | Day 4 | Day 5 | Day 6 | Day 7 |
| --- | --- | --- | --- | --- | --- | --- | --- |
| Amorolfine 10% | 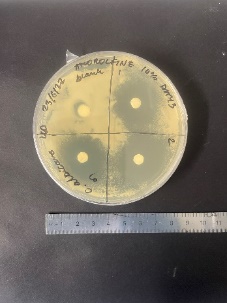 | 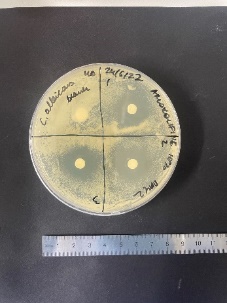 | 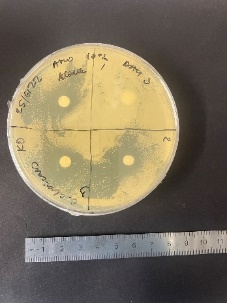 | 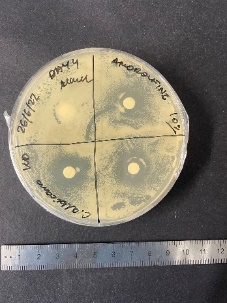 | 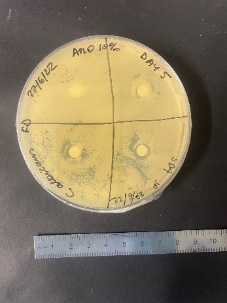 | 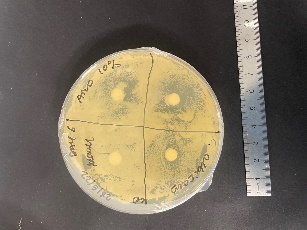 | 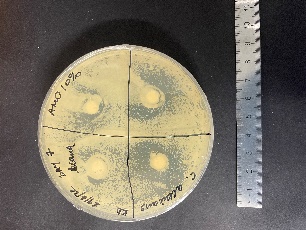 |
| Terbinafine 10% | 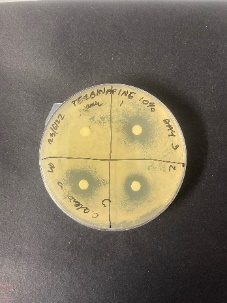 | 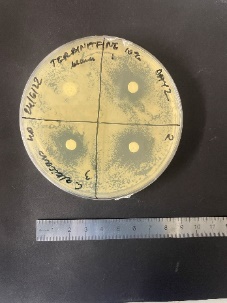 | 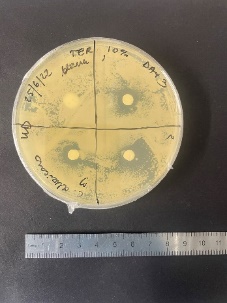 | 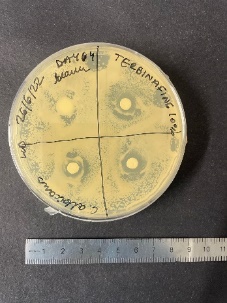 | 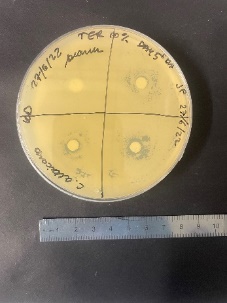 | 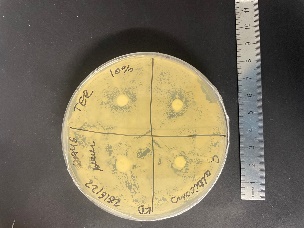 | 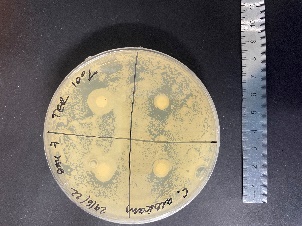 |
| Mix 10% | 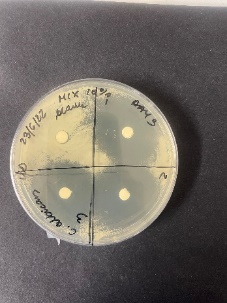 | 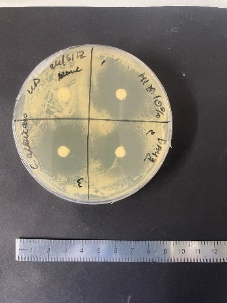 | 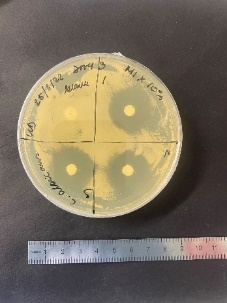 | 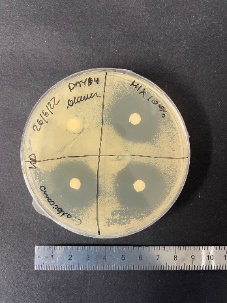 | 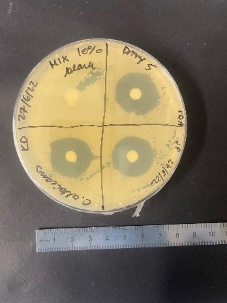 | 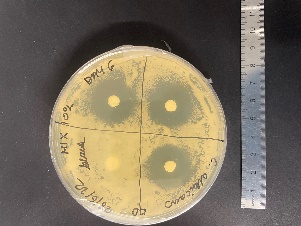 | 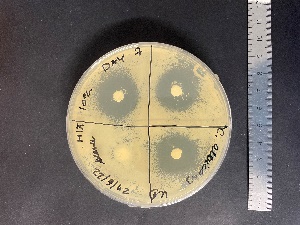 |
